# Antilymphocyte Antibodies in Systemic Lupus Erythematosus: Association with Disease Activity and Lymphopenia

**DOI:** 10.1155/2014/672126

**Published:** 2014-04-17

**Authors:** Chun Li, Rong Mu, Xiao-yan Lu, Jing He, Ru-lin Jia, Zhan-guo Li

**Affiliations:** ^1^Department of Rheumatology and Immunology, Peking University People's Hospital, 11 South Xizhimen Street, Beijing 100044, China; ^2^Department of Rheumatology and Immunology, Peking University Shenzhen Hospital, 1120 Futianlianhua Road, Shenzhen 518036, China

## Abstract

*Purpose*. We analyzed the prevalence, clinical correlation, and the functional significance of ALA in patients with systemic lupus erythematosus (SLE). *Methods*. ALA IgG was detected by indirect immunofluorescence in the serum of 130 SLE patients, 75 patients with various rheumatic diseases, and 45 healthy controls (HC). *Results*. The sensitivity and specificity of ALA IgG in SLE were 42.3% and 96.7%, respectively. ALA was observed in 55.6% (50/90) of patients with lymphopenia, which was significantly higher than in patients with normal lymphocytes (5/40, 12.5%; *P* < 0.001). Patients with active SLE showed higher ALA positivity (60.9%) than those with inactive disease (24.2%; *χ* ^2^ = 17.925; *P* < 0.001). ALA correlated significantly with hypocomplementemia, anti-dsDNA antibodies, and higher SLEDAI scores. The incidences of ALA in SLE patients who were seronegative for anti-dsDNA, anti-Sm, or both antibodies were 32.9% (26/79), 41.0% (43/105), and 32.4% (22/68), respectively. The ALA-positive group also had higher incidences of neuropsychiatric SLE (NPSLE) and lupus nephritis (LN). In multivariate analyses, ALA was independently associated with lymphopenia, higher SLEDAI scores, and increased risk for LN. ALA titers significantly decreased as clinical disease was ameliorated following treatment. *Conclusions*. ALA occurred more frequently in patients with active SLE and was independently associated with lymphopenia, disease activity, and LN.

## 1. Introduction

Lymphopenia is a common clinical manifestation in systemic lupus erythematosus (SLE) and is one of the diagnostic criteria, according to the American College of Rheumatology (ACR) classification [1]. Lymphopenia was observed in 62% of adult patients at the diagnosis of SLE [2]. The cumulative percentage of the occurrence of lymphopenia over the course of the disease reached over 90% in an adult series [2]. In addition to its clinical use as a diagnostic marker, lymphopenia is associated with disease activity and organ damage [3, 4]. Studies have also suggested that lymphopenia is a risk factor for carotid intima-media thickness in juvenile-onset SLE [5].

Because lymphopenia is a common manifestation in SLE, people have long been interested in antibodies against lymphocytes. Lymphocytotoxic antibodies (LCA) were found in the great majority of patients with SLE [6, 7]. The standard method for the detection of LCA is a microcytotoxicity test. However, subtle differences in the protocol, such as incubation/isolation temperature, method of target cell isolation, and serum dilution, can result in result variability, and this has created many controversies in the literature. A practical indirect immunofluorescence test, for ALA, was developed to overcome the shortcomings of LCA. LCA was shown to be cold-reactive and to detect IgM exclusively [6, 8, 9]. However, Agnello suggested that the IgG type may be more important functionally [10] and more effective at physiological temperatures.

Despite this, there has been little research investigating the role of ALA IgG in SLE. The causal relationship between ALA and lymphocyte function is also largely unexplored. In the present study, we detected ALA IgG and analyzed the possibility of using ALA IgG as a biomarker for disease activity in SLE. We also explored the relationship between lymphopenia and ALA.

## 2. Materials and Methods

### 2.1. Patients

In total, 130 Chinese SLE patients who attended to the Department of Rheumatology and Immunology, Peking University People's Hospital, were enrolled. All patients were on stable doses of glucocorticoids in the previous month and did not used immunosuppressants in the previous 6 months. Serum samples were obtained from the patients. All patients fulfilled the 1997 revised American College of Rheumatology SLE criteria [1].

Also, 75 patients with other autoimmune diseases were used as a disease control group. There were 16 Sjögren's syndrome (SS), 21 rheumatoid arthritis (RA), 16 ankylosing spondylitis (AS), 5 dermatomyositis/polymyositis (DM/PM), 5 undifferentiated connective tissue diseases (UCTD), 7 systemic sclerosis (SSc), and 5 osteoarthritis (OA) cases. In these control patients, there were 56 females and 19 males (female : male = 2.95 : 1). Healthy control (HC) serum samples were obtained from 45 blood donors. All sera were kept at −20°C.

The protocol for the study was approved by the Ethical Committee of Peking University People's Hospital (FWA00001384).

### 2.2. Methods

#### 2.2.1. Detection of Antilymphocyte Antibody (ALA)

Serum ALA was assayed using an indirect immunofluorescence test kit according to the manufacturer's protocol (EUROIMMUN, Germany).

#### 2.2.2. Identification of ALA

The identification of ALA included two steps to ensure the specificity. First, ANCA were detected by an indirect immunofluorescence test (EUROIMMUN) to exclude cross-reactivity. Second, Biochip slides were incubated with RNase-free DNase RQ1 (1 U/*μ*L; TaKaRa Biotechnology, Dalian, China) at room temperature. Control slides were treated with trypsin (0.125 *μ*g/µL, Dingguo, Beijing) for 30 min at 37°C. Then, 5 *μ*L DNase RQ1 was added to serum samples in a final volume of 50 *μ*L and incubated for 60 min at room temperature. Also, 5 *μ*L enzyme-free buffer was used as a control. The reaction was stopped by adding 5 *μ*L 0.5 Methylenediaminetetraacetic acid (EDTA). After washing the slides twice in PBS, subsequent steps were performed according to the manufacturer's protocol.

#### 2.2.3. Clinical and Laboratory Parameters

Clinical and laboratory features of SLE patients were recorded. Lymphopenia was defined according to the ACR criteria (<1.5 × 10^9^/L) and was scored only if physicians determined that it was attributed to SLE and not to medications or other causes. Leucopenia was defined as a white blood cell (WBC) count <4 × 10^9^/L. The “systemic lupus erythematosus disease activity index” (SLEDAI) was used to assess disease activity. A SLEDAI score > 8 was defined as active lupus, as described previously [11].

### 2.3. Statistical Analysis

Data with a normal distribution are expressed as the mean ± SD. Nonnormally distributed data are expressed as the median and interquartile range. Categorical variables were analyzed using the *χ* ^2^ test or Fisher's exact test. Student's *t*-test or analysis of variance (ANOVA) was used for continuous data and the Mann-Whitney *U*-test was used for nonnormally distributed data. The ALA titers before and after treatment were compared using Wilcoxon's signed rank test. Multivariate analysis was performed to investigate the relationship between ALA, lymphopenia, the development of LN or NPSLE and SLEDAI scores, adjusting for gender, age, and disease duration. Statistical significance was defined as *P* < 0.05 (two-tailed). All statistical analyses were performed using the SPSS software (ver. 16.0).

## 3. Results

**Figure 1:**
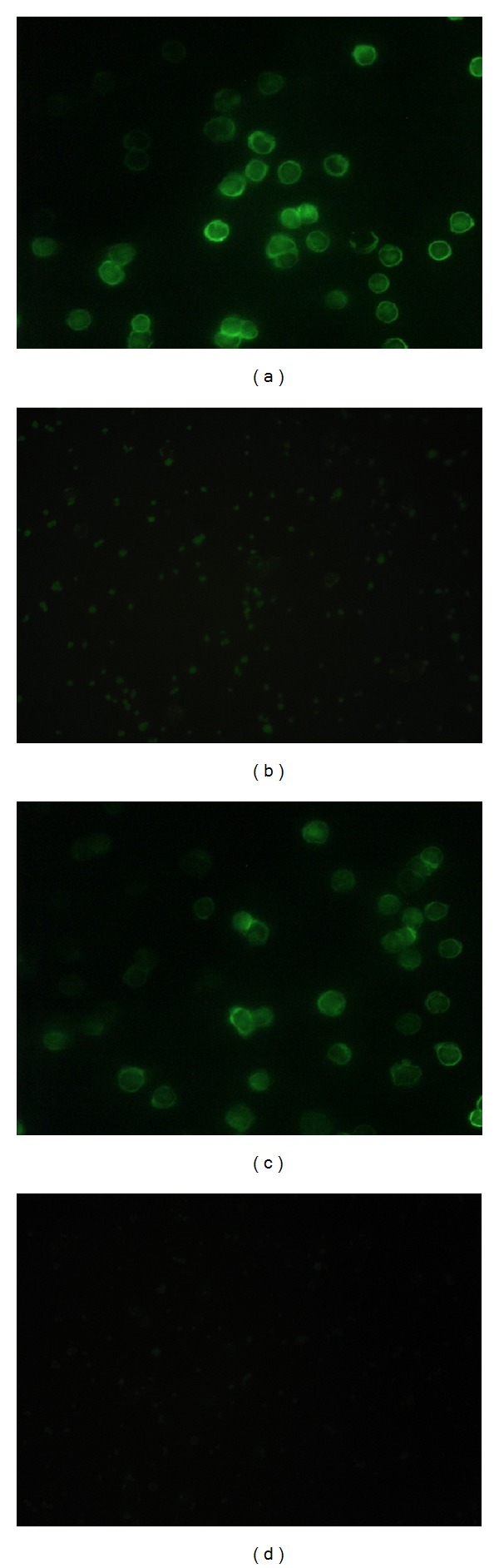
ALA immunofluorescence patterns (×400). (a) ALA-positive pattern with fluorescence in the cytoplasm or a linear annular fluorescence around the lymphocyte cell membrane. (b) ALA-negative pattern with the absence of fluorescence in the cytoplasm or the lymphocyte cell membrane. (c) Immunofluorescence patterns on lymphocytes pretreated with DNase. (d) Immunofluorescence patterns on lymphocytes pretreated with trypsin. This result indicated that the ALA antigen was a protein.

Under microscopic examination, ALA was shown as a fluorescence of the cytoplasm or as a linear annular fluorescence of the lymphocyte cell membrane (Figure 1(a)). For serum without ALA, there was no fluorescence in either the cytoplasm or around the cell membrane (Figure 1(b)). In the identification test, there was no fluorescence in the cytoplasm or linear annular fluorescence in neutrophils or granulocytes on slides. The fluorescence pattern and intensity did not change after pretreatment with RNase-free DNase RQ1 (Figure 1(c)), whereas there was no fluorescence in slides pretreated with trypsin (Figure 1(d)), indicating that the ALA antigen was a protein.

**Figure 2:**
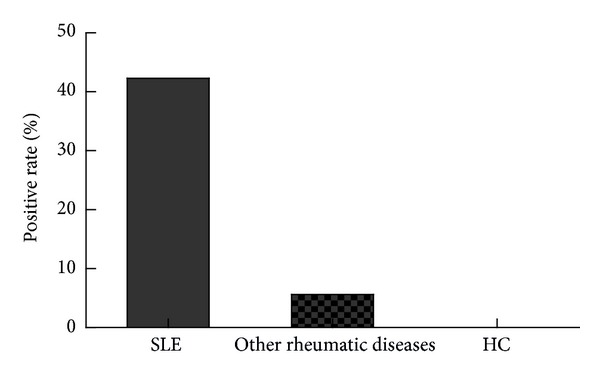
Prevalence of ALA in patients with SLE. The positive rate of ALA in patients with SLE was significantly higher than the other conditions in the control group (4/75, 5.6%): DM/PM (1/5, 20%), SS (2/16, 12.5%), RA (1/21, 4.8%), UCTD (0/5, 0%), SSc (0/7, 0%), OA (0/5, 0%), AS (0/16, 0%), and HC (0/45, 0%), respectively (*P* < 0.001).

Of the 130 SLE patients, 55 (42.3%) were positive for ALA. No healthy control was positive for ALA (*P* < 0.001; Table 1). The prevalence in other rheumatic diseases was significantly lower at 5.6% (4/72, *P* < 0.001, Figure 2). The sensitivity and specificity of ALA for the diagnosis of SLE were 42.3% and 96.7%, respectively.

The positive rate of ALA in the lymphopenia group was much higher than in the normal lymphocyte group (55.6% versus 12.5%; *P* < 0.001). The prevalences of lupus nephritis (76.4%) and NPSLE (18.2%) in ALA-positive SLE patients were significantly higher than in ALA-negative SLE patients (29.3% and 4%, resp.; *P* < 0.05) (Table 2). The SLEDAI score of the ALA-positive group was 11.84 ± 5.36, compared with 8.33 ± 5.71 in the negative group (*P* < 0.05). Using multivariate logistic regression, adjusting for gender, age, and disease duration, ALA was independently associated with lymphopenia (odd ratio (OR) 6.034, 95% confidence interval (CI) 1.385–26.301; *P* = 0.017), disease activity (OR 3.713, 95% CI 1.560–8.835; *P* = 0.003), and increased risk for LN (OR 5.873, 95% CI 2.523–13.672; *P* < 0.001), but not for NPSLE (OR 4.495, 95% CI 0.969–20.865; *P* = 0.055).

Serum complement (C3, C4) levels were much lower in ALA-positive versus ALA-negative patients (*P* < 0.05; Table 3). The frequencies of ANA and anti-dsDNA antibodies were significantly higher in ALA-positive SLE patients (94.5% and 50.9%) than in the ALA-negative group (80% versus 29.3%, *P* < 0.05) (Table 3).

ALA was also found in some anti-dsDNA and anti-Sm-negative patients. The positive rates of ALA were 32.9%, 41.0%, and 32.4% in anti-dsDNA negative, anti-Sm negative, and double-negative SLE patients, respectively.

**Figure 3:**
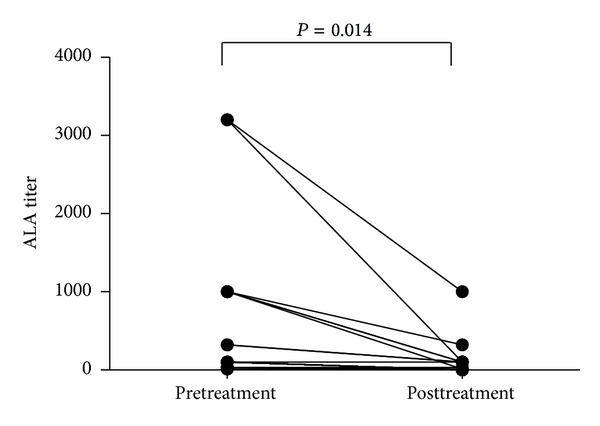
ALA titers before and after treatment in 20 patients with SLE. The ALA titers decreased significantly in accordance with clinical amelioration of disease following treatment (*P* = 0.014).

ALA titers decreased significantly in accordance with disease amelioration following treatment in a subgroup of 20 patients with SLE (Figure 3). The SLEDAI scores in these 20 patients also decreased from 15.8 ± 6.2 to 3.6 ± 3.0 (*P* < 0.001).

## 4. Discussion

Our study demonstrated that the sensitivity and specificity of ALA IgG in SLE were 42.3% and 96.7%, respectively. ALA was independently associated with lymphopenia. The SLEDAI scores and the prevalences of LN and NPSLE were significantly higher in ALA-positive SLE patients than in ALA-negative SLE patients. ALA was also associated with disease activity and LN. Multivariate analysis revealed that ALA was independently associated with disease activity and increased risk for LN. ALA titers decreased significantly in accordance with disease amelioration and monitoring the titer of this antibody may be helpful for predicting disease flares.

As suggested by Magalhães et al., LCA in SLE is associated with disease activity, regardless of the presence of neuropsychiatric manifestations [12]. In the present study, we also found that ALA was associated with disease activity parameters, such as hypocomplementemia, anti-dsDNA antibody, and SLEDAI scores, confirming that ALA is a meaningful biomarker for disease activity (Table 3). Furthermore, ALA was related to anti-dsDNA antibody; however, it was also seen in 32.9% of anti-dsDNA-negative SLE patients. Thus, ALA may be a better or supplementary parameter of disease activity.

ALA is believed to act in both cryoprecipitate formation and the development of organ damage [13, 14]. Our results are consistent with previous studies showing that ALA was correlated with organ involvement, such as LN, indicating that ALA is a predictor for poor prognosis and may play a role in the pathogenesis of SLE. Osman and Swaak's study showed an overlap between ALA and anti-*β*2-microglobulin [15]. ALA may have an impact on T cells as well as on B-cell function and play a role in LN [16]. Although complete information concerning the molecules with which ALA interacts is not yet available, possible target antigens of ALA include CD45, T-cell receptors, *β*2-microglobulin, and HLA I/HLA II antigens [17].

To date, no direct relationship between ALA and lymphopenia has been reported. In our study, ALA was present in more than half of the SLE patients with lymphopenia. Of the patients with ALA, 90.9% had lymphopenia. In a multivariate analysis, ALA was independently associated with lymphopenia. The results suggest that ALA might be one of the reasons for lymphopenia. Possible mechanisms include the depletion of circulating T cells and ALA may have the capacity for direct actions on target cells, including complement-dependent cytotoxicity and ADCC, modulation of surface antigens, and up- or downregulation of various cell functions in the immune response [15]. Other possible explanations for lymphopenia have been proposed. Several other autoantibodies, such as anti-SSA antibody, anti-snRNP antibody, and anti-dsDNA antibody, may have lymphocytotoxic properties. Another contributing factor to lymphopenia involves defective CD95/Fas systems [18] and diminished expression of complement regulatory proteins (CD55 and CD59) [19]. Despite these hypotheses, our results indicate that ALA plays a role in the pathogenesis of lymphopenia in SLE patients.

## 5. Conclusions

In conclusion, ALA IgG is a good parameter of disease activity and is associated with LN in SLE. The presence of ALA may be important in the mechanism of lymphopenia in SLE.

## Figures and Tables

**Table 1:** Demographic, clinical and laboratory features of SLE patients.

Characteristics	
Female, *n* (%)	122 (93.8)
Age, mean ± SD years	33.3 ± 12.4
Disease duration, median (interquartile range)	2 (0.8–5)
SLEDAI, mean ± SD	9.8 ± 5.8
Clinical features, *n* (%)	
Lupus nephritis	64 (49.2)
NPSLE	13 (10)
Arthralgia	45 (34.6)
Serositis	15 (11.5)
Anti-dsDNA positive, *n* (%)	50 (38.5)
ALA, *n* (%)	55 (42.3)

**Table 2:** The correlation of ALA and clinical manifestations in SLE.

Clinical Features	ALA negative (*N* = 75)		ALA positive (*N* = 55)		*χ* ^ 2^	*P* values
	*n *	%	*n *	%		
LN	22	29.3	42	76.4	28.080	0.000^**※**^
NPSLE	3	4.0	10	18.2	7.091	0.008^**※**^
Skin Rash	35	46.7	27	49.1	0.075	0.785
Photosensitivity	8	10.7	2	3.6	1.330	0.189
Oral Ulcer	10	13.3	8	14.5	0.039	0.843
Arthralgia	29	38.7	16	29.1	1.286	0.257
Serositis	7	9.3	8	14.5	0.845	0.358
Raynaud Phenomenon	6	8.0	6	10.9	0.320	0.571
Alopecia	21	28	18	32.7	0.338	0.561
Myositis	5	6.7	1	1.8	1.694	0.193

^*※*^ *P* < 0.05.

**Table 3:** The relation between ALA and other laboratory parameters in SLE.

Laboratory parameters	ALA negative (*N* = 75)		ALA positive (*N* = 55)		*χ* ^ 2^	*P* values
	*n *	%	*n *	%		
ANA	60	80	52	94.5	5.628	0.018^**※**^
Anti-dsDNA	22	29.3	28	50.9	6.241	0.012^**※**^
Anti-SSA	20	26.7	22	40.0	2.579	0.108
Anti-SSB	5	6.7	6	10.9	0.737	0.391
Anti-RNP	14	18.7	11	20.0	0.036	0.849
Anti-Sm	10	13.3	7	12.7	0.010	0.919
ESR Elevation	50	66.7	45	81.8	3.702	0.054
CRP Elevation	27	36	17	30.9	0.367	0.544
C3 Decrease	58	77.3	54	98.2	11.562	0.001^*※*^
C4 Decrease	51	68	48	87.3	6.490	0.011^*※*^
IgA Elevation	13	17.3	14	25.5	1.272	0.259
IgG Elevation	34	45.3	29	52.7	0.695	0.405
IgM Elevation	5	6.7	5	9.1	0.263	0.608
Leucopenia	31	41.3	32	58.2	3.606	0.058
Thrombocytopenia	16	21.3	8	14.5	0.971	0.324

^*※*^ *P* < 0.05.
